# HIF-1α and TAZ serve as reciprocal co-activators in human breast cancer cells

**DOI:** 10.18632/oncotarget.4190

**Published:** 2015-05-19

**Authors:** Lisha Xiang, Daniele M. Gilkes, Hongxia Hu, Weibo Luo, John W. Bullen, Houjie Liang, Gregg L. Semenza

**Affiliations:** ^1^ Department of Oncology and Southwest Cancer Center, Southwest Hospital, Third Military Medical University, Chongqing, China; ^2^ Vascular Program, Institute for Cell Engineering, Johns Hopkins University School of Medicine, Baltimore, MD, USA; ^3^ McKusick-Nathans Institute of Genetic Medicine, Johns Hopkins University School of Medicine, Baltimore, MD, USA; ^4^ Departments of Pediatrics, Medicine, Oncology, Radiation Oncology, and Biological Chemistry, Johns Hopkins University School of Medicine, Baltimore, MD, USA

**Keywords:** breast cancer progression, hypoxia-inducible factor 1, MDA-MB-231 cells, MCF-7 cells

## Abstract

Hypoxia-inducible factor 1α (HIF-1α) expression is a hallmark of intratumoral hypoxia that is associated with breast cancer metastasis and patient mortality. Previously, we demonstrated that HIF-1 stimulates the expression and activity of TAZ, which is a transcriptional effector of the Hippo signaling pathway, by increasing TAZ synthesis and nuclear localization. Here, we report that direct protein-protein interaction between HIF-1α and TAZ has reciprocal effects: HIF-1α stimulates transactivation mediated by TAZ and TAZ stimulates transactivation mediated by HIF-1α. Inhibition of TAZ expression impairs the hypoxic induction of HIF-1 target genes, such as *PDK1, LDHA, BNIP3* and *P4HA2* in response to hypoxia, whereas inhibition of HIF-1α expression impairs TAZ-mediated transactivation of the *CTGF* promoter. Taken together, these results complement our previous findings and establish bidirectional crosstalk between HIF-1α and TAZ that increases their transcriptional activities in hypoxic cells.

## INTRODUCTION

Intratumoral hypoxia is a critical microenvironmental factor driving breast cancer progression [1, 2]. In response to reduced O_2_ availability, hypoxia-inducible factor 1 (HIF-1) mediates the transcriptional activation of genes encoding proteins that are required for many important steps in breast cancer progression, such as angiogenesis, cancer stem cell maintenance, cell motility, epithelial-mesenchymal transition, extracellular matrix remodeling, metabolic reprogramming, metastasis, and resistance to therapy [3-6]. HIF-1 is a heterodimeric transcription factor, which consists of two subunits, HIF-1α and HIF-1β [7]. In normoxic cells, HIF-1α is hydroxylated by prolyl hydroxylases (PHD1-3) at proline residue 402 and/or 564, and by an asparaginyl hydroxylase (FIH-1) at asparagine residue 803 [reviewed in ref. 8]. The von Hippel-Lindau tumor suppressor protein binds to prolyl hydroxylated HIF-1α and recruits an E3 ubiquitin ligase complex containing Elongin C, Elongin B, and Cullin 2 that ubiquitinates HIF-1α and thereby targets it for degradation by the 26*S* proteasome. In addition, asparaginyl hydroxylation of HIF-1α blocks its binding to the transcriptional co-activator p300 and reduces HIF-1 transcriptional activity. Under hypoxic conditions, prolyl and asparaginyl hydroxylation are inhibited, which stabilizes the HIF-1α protein and promotes HIF-1-dependent gene transcription [reviewed in ref. 8].

A growing number of proteins have been identified that function as co-activators of HIF-1 by binding to HIF-1α and regulating its transcriptional activity. The histone acetyltransferase p300 catalyzes the acetylation of lysine residues on the N-terminal tail of core histones at HIF-1 target genes, leading to changes in chromatin structure that promote HIF-1-dependent gene transcription [9]. Pyruvate kinase M2 (PKM2) also interacts with HIF-1α in the cell nucleus and functions as a transcriptional co-activator in HeLa cervical carcinoma and Hep3B hepatoblastoma cells [10]. PKM2 increased HIF-1 binding to hypoxia response elements (HREs) at target genes, recruitment of p300, histone acetylation, and subsequent transactivation of HIF-1 target genes including *SLC2A1*, *LDHA*, and *PDK1*, which encode glucose transporter 1, lactate dehydrogenase A, and pyruvate dehydrogenase kinase 1, respectively [10]. HIF-1α also recruits histone demethylases including JMJD2C, which demethylates trimethylated lysine 9 of histone H3 and enhances HIF-1 binding to HREs to activate transcription of HIF-1 target genes and drive breast cancer metastasis [11]. Other co-activators of HIF-1 that promote breast cancer progression include Pontin [12] and XBP1s [13].

We reported recently that HIF-1, but not HIF-2, binds directly to the *WWTR1* gene encoding the transcriptional co-activator with PDZ-binding motif (TAZ), an effector of Hippo signaling [5]. TAZ interacts with DNA binding proteins of the TEA/ATTS domain (TEAD) family to activate transcription of target genes, such as *CTGF* (encoding connective tissue growth factor), which promote epithelial-mesenchymal transition and the breast cancer stem-cell phenotype [14-18]. In addition to increasing TAZ mRNA and protein expression, HIF-1 transactivates the *SIAH1* gene, which encodes an ubiquitin protein ligase that is required for the proteasomal degradation of LATS2, a kinase that phosphorylates TAZ and inhibits its nuclear localization [5]. Thus, HIF-1 coordinately regulates TAZ expression and TAZ/TEAD transcriptional activity. HIF-1-dependent TAZ activity induces the breast cancer stem cell phenotype in response to hypoxia [5].

TAZ was reported to interact with HIF-1α and positively regulate HIF-1 transcriptional activity in a breast cancer cell line that was selected for metastasis to bone [18]. Here, we report that TAZ and HIF-1α interact in MDA-MB-231 cells, which metastasize to lymph nodes and lungs in a HIF-dependent manner [19, 20]. Remarkably, we also report that the interaction of HIF-1α with TAZ also stimulates TAZ/TEAD transcriptional activity. Thus, bidirectional functional interactions between HIF-1α and TAZ synergistically drive the expression of downstream target genes in hypoxic breast cancer cells.

## RESULTS

### TAZ serves as a co-activator for HIF-1α

**Figure 1:**
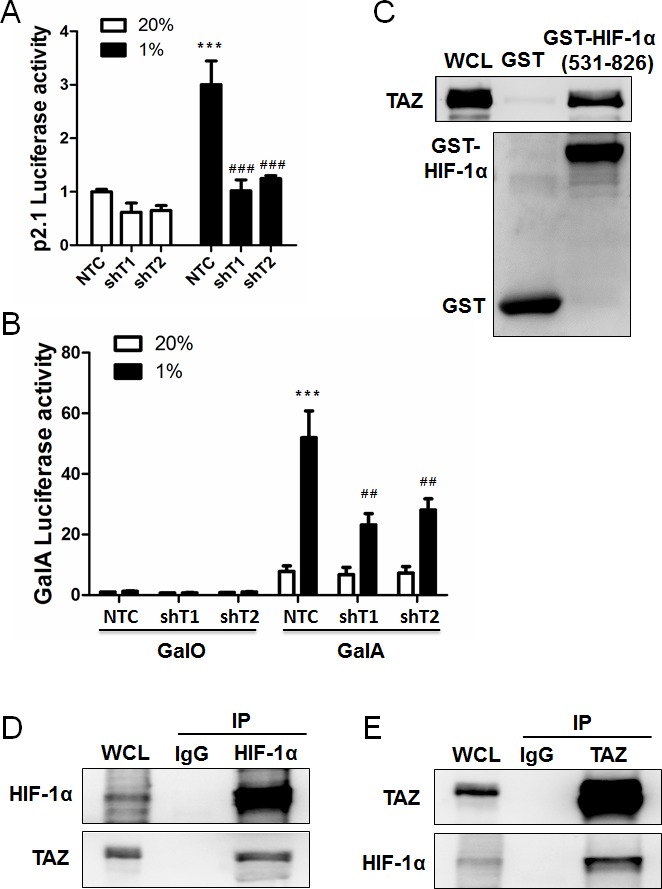
TAZ serves a co-activator for HIF-1α **A.** MDA-MB-231 cells were stably transduced with a lentiviral vector encoding shRNA targeting TAZ (shT1 or shT2) or a non-targeting control shRNA (NTC). These subclones were transiently co-transfected with HIF-1–dependent firefly luciferase reporter plasmid p2.1 and control Renilla luciferase reporter plasmid pSV-Renilla, and exposed to 20% or 1% O_2_ for 24 h. The ratio of firefly:Renilla luciferase activity was normalized to lane 1 (mean ± SEM, *n* = 4). ^***^ *P* < 0.001 versus NTC at 20% O_2_; ^###^*P* < 0.001 versus NTC at 1% O_2_. **B.** MDA-MB-231 subclones were transiently co-transfected with expression vector pGalO (encoding GAL4 DNA-binding domain) or pGalA (encoding GAL4 DNA-binding domain fused to amino acid residues 531-826 of HIF-1α), GAL4-dependent firefly-luciferase reporter-plasmid pG5E1bLuc, and pSV-Renilla. Transfected cells were exposed to 20% or 1% O_2_ for 24 h. The luciferase activity was normalized to lane 1 (mean ± SEM, *n* = 4). ^***^P < 0.001 versus NTC at 20% O_2_; ^##^*P* < 0.01 versus NTC at 1% O_2_. **C.** GST pull-down assays were performed with GST or a GST fusion protein containing residues 531-826 of HIF-1α and whole cell lysate (WCL) from MDA-MB-231 cells that were exposed to 1% O_2_ for 24 h. Immunoblot assays were performed with antibodies against TAZ (upper panel) and GST (lower panel). **D-E**. Immunoprecipitation was performed with anti-HIF-1α **(D)**, anti-TAZ **(E)**, or IgG (D and E) antibodies and WCL from MDA-MB-231 cells that were exposed to 1% O_2_ for 4 h. The immunoprecipitates were analyzed by immunoblot assays using antibodies against HIF-1α and TAZ.

We previously established MDA-MB-231 subclones, which were stably transduced with a lentiviral expression vector encoding a non-targeting control (NTC) short hairpin RNA (shRNA) or either of two independent shRNAs targeting TAZ (shT1 and shT2), and immunoblot assays confirmed effective knockdown of TAZ protein expression, with no effect on HIF-1α protein levels [5]. To determine whether TAZ regulates HIF-1 transcriptional activity, MDA-MB-231 subclones were co-transfected with HIF-1–dependent reporter plasmid p2.1, which contains an HRE from the human *ENO1* gene upstream of SV40 promoter and firefly luciferase coding sequences [21], and control reporter pSV-Renilla, which contains the SV40 promoter upstream of Renilla luciferase coding sequences. The ratio of firefly:Renilla luciferase activity is a measure of HIF-1 transcriptional activity. Knockdown of endogenous TAZ expression significantly reduced HIF-1 transcriptional activity under hypoxic conditions (Figure 1A).

To determine whether TAZ directly regulates HIF-1α transactivation domain (TAD) function, MDA-MB-231 NTC and TAZ-knockdown subclones were co-transfected with the following plasmids: expression vector pGalA [22], which encodes the GAL4 DNA-binding domain fused to the HIF-1α TAD (amino acid residues 531–826) or pGalO, which encodes only the GAL4 DNA-binding domain; reporter plasmid pG5E1bLuc, which contains five GAL4-binding sites and a TATA box from the adenovirus *E1b* gene upstream of firefly luciferase coding sequences; and pSV-Renilla. Exposure of the cells to 1% O_2_ for 24 h increased GalA-dependent, but not GalO-dependent, luciferase activity in MDA-MB-231 cells transfected with NTC vector, whereas GalA-dependent transcription was significantly decreased in cells transfected with vector encoding TAZ shRNA (Figure 1B).

To determine whether TAZ stimulates HIF-1α TAD function by direct interaction, glutathione-*S*-transferase (GST) and a recombinant GST-HIF-1α (531-826) fusion protein were purified from bacteria, incubated with whole cell lysates and glutathione-agarose beads, and the bound proteins were analyzed by immunoblot assays using anti-TAZ and anti-GST antibodies, which revealed binding of TAZ to GST-HIF-1α (531-826) but not to GST alone (Figure 1C). Interaction of endogenous TAZ and HIF-1α in hypoxic MDA-MB-231 cells was also demonstrated by co-immunoprecipitation assays, using anti-HIF-1α antibody to precipitate TAZ (Figure 1D) and anti-TAZ antibody to precipitate HIF-1α (Figure 1E). Taken together, these results demonstrate that TAZ functions as a co-activator for HIF-1-dependent gene transcription.

### HIF-1α serves as a co-activator for TAZ

**Figure 2:**
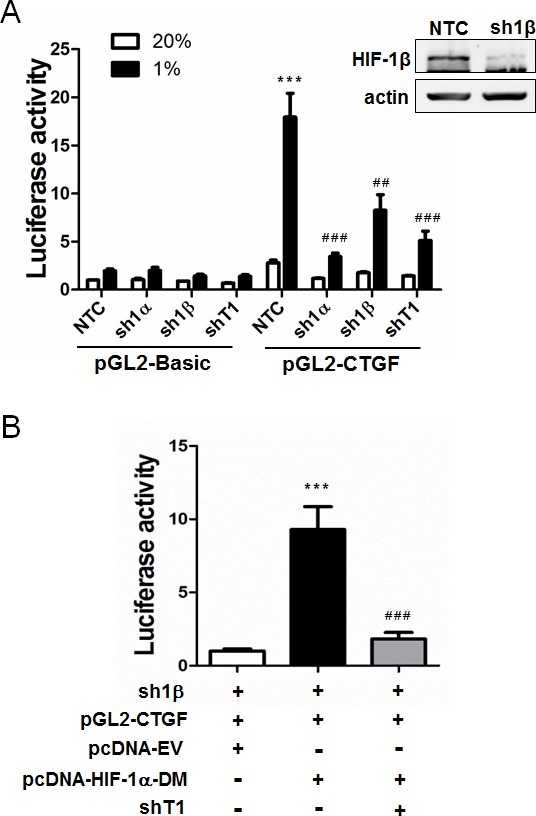
HIF-1α serves as a co-activator for TAZ **A.** MCF-7 cells were stably transfected with a lentiviral expression vector encoding a non-targeting control shRNA (NTC) or shRNA targeting HIF-1α (sh1α), HIF-1β (sh1β; efficiency of knockdown shown in immunoblot at upper right) or TAZ (shT1). The MCF-7 subclones were transiently transfected with pSV-Renilla and either the pGL2-Basic or pGL2-CTGF firefly luciferase reporter, and exposed to 20% or 1% O_2_ for 24 h. The luciferase activity was normalized to lane 1 (mean ± SEM, n = 4). ^***^P < 0.001 versus NTC at 20% O_2_;^##^*P* < 0.01, ^###^*P* < 0.001 versus NTC at 1% O_2_. **B.** MCF-7 cells stably transfected with HIF-1β shRNA (sh1β) vector were transiently transfected with pGL2-CTGF, pSV-Renilla, and either pcDNA-EV (empty vector) or pcDNA-HIF-1α-DM (encoding double mutant HIF-1α [P402A/P564A]) in the presence of TAZ shRNA (shT1) or NTC vector. The luciferase activity was normalized to lane 1 (mean ± SEM, *n* = 4). ^***^P < 0.001 versus lane 1; ^###^*P* < 0.001 versus lane 2.

We next investigated whether HIF-1α regulates TAZ transcriptional activity. We established MCF-7 breast cancer subclones, which were stably transduced with lentiviral vectors encoding NTC shRNA or shRNA targeting HIF-1α (sh1α), HIF-1β (sh1β), or TAZ (shT1), and immunoblot assays confirmed effective knockdown of HIF-1α and TAZ [5] as well as HIF-1β (Figure 2A) protein expression. A 247-bp *CTGF* proximal promoter sequence, which contains three copies of the TEAD-binding site sequence (5′-GGAATG-3′) and no match to the HIF-1 binding site consensus sequence (5′-RCGTG-3′), was inserted into the promoterless firefly luciferase reporter plasmid pGL2-Basic to generate pGL2-CTGF. MCF-7 subclones were transiently transfected with pSV-Renilla and either pGL2-CTGF or pGL2-Basic. Luciferase activity was increased in response to hypoxia in NTC cells transfected with pGL2-CTGF, whereas knockdown of HIF-1α, HIF-1β, or TAZ significantly decreased luciferase activity under hypoxia and the effect of HIF-1α knockdown was greater than the effect of HIF-1β knockdown (Figure 2A).

The loss of *CTGF* promoter reporter activity in hypoxic cells with HIF-1α or HIF-1β knockdown reflected the loss of HIF-1-mediated TAZ mRNA and protein expression [5]. However, the greater inhibitory effect of HIF-1α knockdown suggested that HIF-1α might also directly stimulate TAZ transcriptional activity through its physical interaction with TAZ. To test this hypothesis, we expressed a constitutively active form of HIF-1α (with mutation of the two proline residues that are subject to O_2_-dependent hydroxylation) under non-hypoxic conditions in HIF-1β knockdown MCF-7 cells to eliminate HIF-1-dependent activation of *WWTR1* gene transcription. Under these conditions, *CTGF* promoter activity was still increased by HIF-1α and this effect was lost when TAZ expression was knocked down (Figure 2B), indicating that HIF-1α functions as a co-activator of TAZ-dependent transcription.

### Co-activator functions of TAZ and HIF-1α contribute to the transcription of endogenous target genes

**Figure 3:**
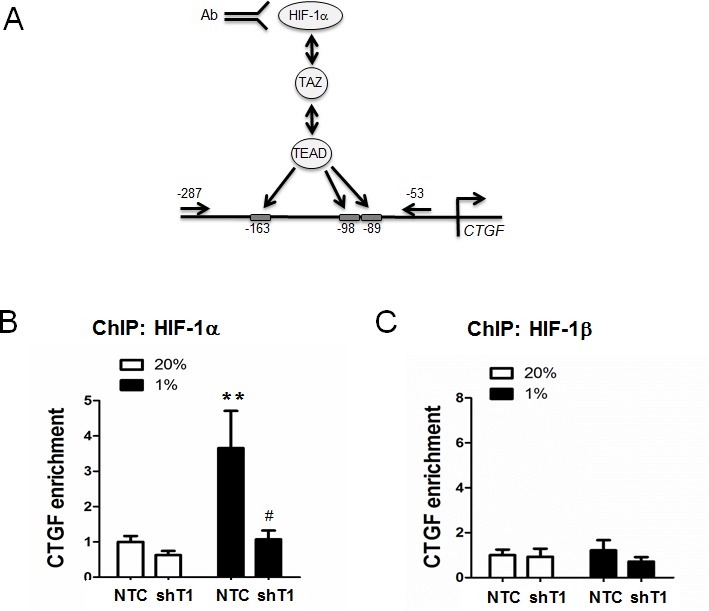
Chromatin immunoprecipitation (ChIP) assays of selective HIF-1α binding to the *CTGF* proximal promoter **A.** Schematic diagram of primers specific for the *CTGF* proximal promoter, which contains 3 TEAD binding sites and no HIF-1 binding sites. **B-C.** MCF-7 subclones (NTC and shT1) were exposed to 20% or 1% O_2_ for 16 h. Chromatin was immunoprecipitated with anti-HIF-1α **(B)** or anti-HIF-1β **(C)** antibody and analyzed by qPCR with ChIP primers shown in panel A (mean ± SEM, *n* =3). ^**^P < 0.01 versus NTC at 20%; ^#^*P* < 0.05 versus NTC at 1% O_2_.

We next investigated whether TAZ and HIF-1α function as reciprocal co-activators in transcription of their endogenous target genes. We reported previously that CTGF mRNA expression increased in MCF-7 cells in response to hypoxia, but induction was lost when expression of HIF-1α or TAZ was silenced by shRNA [5]. To investigate whether TAZ recruits HIF-1α and HIF-1β to the *CTGF* promoter, chromatin immunoprecipitation (ChIP) assays were performed in MCF-7 cells stably transduced with NTC or shT1 expression vector. ChIP primers were designed to amplify the region of the *CTGF* proximal promoter, which was analyzed by reporter assays above, containing three TEAD binding sites and no HIF-1 binding sites (Figure 3A). Prior studies demonstrated that TAZ-dependent transcriptional co-activation of the *CTGF* promoter was TEAD-dependent [14, 15]. Hypoxia induced the binding of HIF-1α (Figure 3B), but not HIF-1β (Figure 3C), to the *CTGF* promoter in NTC cells, but not in TAZ knockdown cells, demonstrating that TAZ recruits HIF-1α to the *CTGF* proximal promoter in hypoxic breast cancer cells.

**Figure 4:**
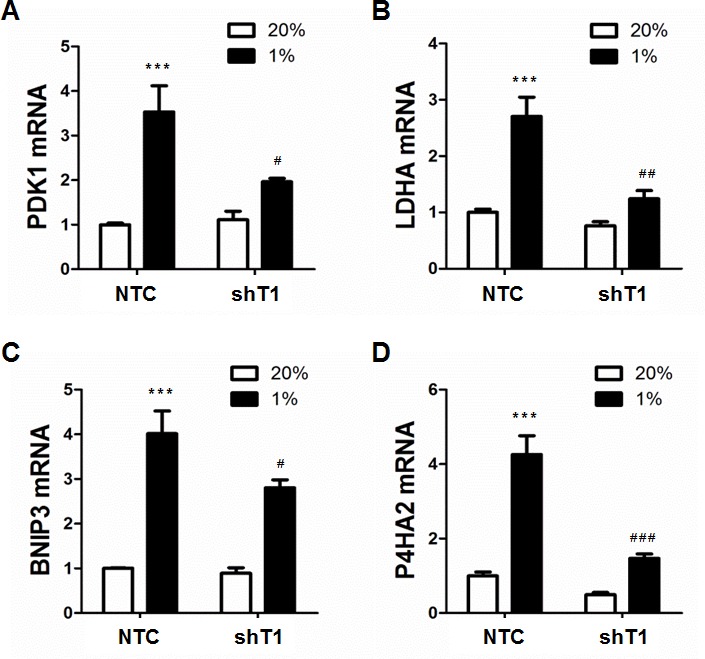
Analysis of HIF-1 target gene expression **A-D.** MDA-MB-231 subclones (NTC and shT1) were exposed to 20% or 1% O_2_ for 24 h and total cellular RNA was isolated. Reverse transcription and qPCR analyses of PDK1 (**A**), LDHA (**B**), BNIP3 (**C**), and P4HA2 (**D**) mRNAs were performed (mean ± SEM, *n* =3). ^***^P < 0.001 versus NTC at 20% O_2_; ^#^*P* < 0.05, ^##^*P* < 0.01, ^###^*P* < 0.001 versus NTC at 1% O_2_.

**Figure 5:**
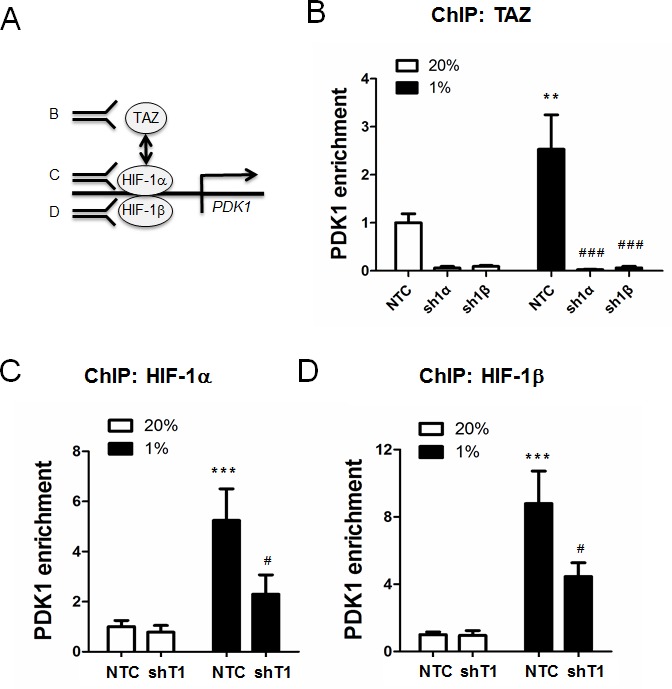
ChIP assays of TAZ and HIF-1 binding to the PDK1 gene in chromatin **A.** ChIP assays were performed using antibodies against TAZ (panel B), HIF-1α (panel C), or HIF-1β (panel D), followed by quantitative real-time PCR with primers spanning the *PDK1* hypoxia-response element (mean ± SEM, *n* =3). **B.** MCF-7 subclones (NTC, sh1α, and sh1β) were exposed to 20% or 1% O_2_ for 16 h. Chromatin was immunoprecipitated with anti-TAZ antibody. ^**^P < 0.01 versus NTC 20% O_2_; ^###^*P* < 0.001 versus NTC at 1% O_2_. (C-D) MCF-7 subclones (NTC and shT1) were exposed to 20% or 1% O_2_ for 16 h. Chromatin was immunoprecipitated with anti-HIF-1α **(C)** or anti-HIF-1β **(D)** antibody. ^***^P < 0.001 versus NTC at 20% O_2_; ^#^*P* < 0.05 versus NTC at 1% O_2_.

To determine whether TAZ regulates the expression of HIF-1 target genes, MDA-MB-231 cells, which were stably transduced with NTC or shT1 vector, were exposed to 20% or 1% O_2_ for 24 h and total RNA was isolated. TAZ knockdown significantly decreased the hypoxia-induced expression of the HIF-1 target genes *PDK1* (Figure 4A), *LDHA* (Figure 4B), *BNIP3* (Figure 4C) and *P4HA2* (Figure 4D), whereas TAZ knockdown had no significant effect on the expression of these genes in non-hypoxic cells (Figure 4A-4D). ChIP assays revealed hypoxia-induced binding of TAZ to the HRE located in the 5′-flanking region of the *PDK1* gene, which was abrogated by knockdown of HIF-1α or HIF-1β (Figure 5A). In addition, hypoxia-induced binding of HIF-1α (Figure 5B) and HIF-1β (Figure 5C) to the HRE in the *PDK1* gene was significantly decreased in TAZ knockdown cells. Taken together, the data presented in Figures 3, 4, 5 demonstrate that HIF-1α functions as a co-activator for the transcription of TAZ/TEAD target genes and TAZ functions as a co-activator for the transcription of HIF-1 target genes in hypoxic human breast cancer cells.

## DISCUSSION

HIF-1α plays critical roles in tumor angiogenesis and metabolic reprogramming, as well as multiple steps in the process of breast cancer invasion and metastasis [3, 23]. HIF-1 and TAZ/TEAD transcriptional activity appear to play important roles in driving breast cancer progression, particularly maintenance of the breast cancer stem cell phenotype [5, 16]. In a previous study, we demonstrated that under hypoxic conditions, HIF-1 increased transcription of the *WWTR1* gene encoding TAZ and the *SIAH1* gene encoding a ubiquitin ligase that triggers degradation of LATS2, a negative regulator of TAZ nuclear localization in breast cancer cells [5]. HIF-1 was also reported to transactivate the *SIAH2* gene to degrade LATS2 and stabilize YAP, which is a TAZ homolog that also serves as a co-activator for TEAD DNA-binding proteins in the Hippo pathway [24].

In the present study, we demonstrate that HIF-1α physically interacts with TAZ and serves as a co-activator for TEAD/TAZ-dependent transcription, providing a novel molecular mechanism by HIF-1α stimulates transcription of the *CTGF* gene, which has been shown to be directly transactivated by either TAZ/TEAD or HIF-1 previously [5, 14, 15, 25]. Thus, HIF-1α may regulate *CTGF* both as a transcriptional activator (after heterodimerization with HIF-1β and binding to an HRE in the distal promoter) or as a co-activator (by interacting with the TAZ/TEAD complex bound to the proximal promoter). Many other genes are known to be co-regulated by HIF-1 and TAZ, including those encoding plasminogen activator inhibitor 1 and the anti-apoptotic factor survivin [5]. Further studies are required to determine whether HIF-1 also regulates those genes both directly, as a transcriptional activator, and indirectly as a TAZ/TEAD co-activator.

HIF-1α and TAZ may also complement each other by activating different genes that function in the same pathway. For example, both HIF-1α and TAZ play key roles in remodeling of the extracellular matrix, which is critical for tissue invasion [26, 27]. We previously demonstrated that HIF-1α and TAZ cooperate to induce the breast cancer stem cell phenotype [5] but further studies are required to determine the specific target genes and mechanisms of transcriptional activation and co-activation that mediate this effect.

We also show that the TAZ-HIF-1α interaction stimulates HIF-1 target gene expression, such that TAZ and HIF-1α function as reciprocal co-activators. A previous study reported that TAZ served as a HIF-1 co-activator in breast cancer cells selected for metastasis to bone [18], whereas our study expands this co-activator function to MDA-MB-231 breast cancer cells, which metastasize to lungs and lymph nodes, as well as non-metastatic MCF-7 cells. We demonstrate that one of the mechanisms by which TAZ stimulates HIF-1 transcriptional activity is by stabilizing its binding to hypoxia response elements (Figure 5C-5D), which has been observed for other HIF-1 co-activators, including JMJD2C and PKM2 [10, 11].

**Figure 6:**
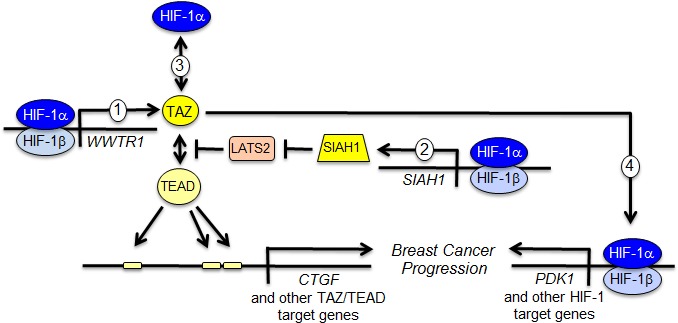
Reciprocal crosstalk promotes breast cancer progression The schematic summarizes four distinct mechanisms of functional interaction between HIF-1α and TAZ as determined in this study and previous work [5, 14]. (1) HIF-1 activates transcription of the *WWTR1* gene encoding TAZ. (2) HIF-1 activates transcription of the *SIAH1* gene encoding an ubiquitin protein ligase that mediates the degradation of LATS2, a negative regulator of TAZ nuclear localization. (3) HIF-1α interacts with TAZ to stimulate transcription of TAZ/TEAD target genes such as *CTGF*. (4) TAZ interacts with HIF-1α to stimulate transcription of HIF-1 target genes such as *PDK1.*

Taken together with our previous study [5], we have now identified four discrete mechanisms of crosstalk between HIF-1α and TAZ that serve to increase the activity of both pathways (Figure 6) and drive breast cancer progression [5]. Increased expression of both TAZ and HIF-1 target genes in primary breast tumors is associated with increased patient mortality [5], underscoring the clinical consequences of functional interactions between these two transcriptional regulators. Therapeutic strategies that include drugs that inhibit HIF-1α protein accumulation [19, 20, 28] or perhaps TAZ target gene products [29] may improve outcome in women with triple negative breast cancer, who are currently treated with cytotoxic chemotherapy with durable response rates of less than 20%.

## MATERIALS AND METHODS

### Cell culture

Breast cancer cell lines MDA-MB-231 and MCF-7 were cultured in high-glucose (4.5 mg/mL) DMEM supplemented with 10% (v/v) fetal bovine serum and 1% (v/v) penicillin-streptomycin (Invitrogen). All cells were maintained at 37°C in a 5% CO_2_, 95% (v/v) air incubator. For hypoxic exposure, cells were placed in a modular incubator chamber (Billups–Rothenberg) and flushed with a 1% O_2_/5% CO_2_/94% N_2_ (v/v) gas mixture.

### ShRNA, lentiviruses, and transduction

Vectors encoding shRNA targeting HIF-1α and TAZ were previously described [5]. The sense strand of the 21-nucleotide sequence encoding shRNA targeting HIF-1β was 5′-GCCTACACTCTCCAACACAAT-3′. Oligonucleotides were annealed and ligated into *Age*I/*Eco*RI-linearized pLKO.1-puro lentiviral vector. The lentiviral vectors were co-transfected with plasmid pCMV-dR8.91 and a plasmid encoding vesicular stomatitis virus G protein into 293-T cells using Lipofectamine 2000 (Invitrogen). Medium containing viral particles was collected 48 h after transfection and passed through a 0.45-μM filter. MDA-MB-231 and MCF-7 cells were transduced with viral supernatant supplemented with 8 μg/mL Polybrene (Sigma-Aldrich). After 24 h, cells were selected in medium containing 0.6 μg/mL puromycin (Sigma-Aldrich).

### Luciferase reporter plasmid constructs

A 247-bp *CTGF* proximal promoter sequence was amplified from human genomic DNA by PCR (primers: 5′-CCCCTCGAGAGTGTGCCAGCTTTTTCAGAC-3′ and 5′-CGAAGCTTCGAGCTGGAGGGTGGAGT-3′), purified by gel extraction, and inserted into the *Xho*I and *Hin*dIII sites of pGL2-Basic (Promega). Plasmid constructs were confirmed by nucleotide sequencing.

### Reverse transcription and real-time quantitative PCR (qPCR)

Total cellular RNA was extracted using TRIzol (Invitrogen), precipitated with isopropanol, treated with DNase I (Ambion), and reverse transcribed using the iScript cDNA Synthesis kit (Bio-Rad). qPCR analysis was performed using Maxima SYBR Green Master Mix (Fermentas) with the iCycler Real-time PCR Detection System (BioRad). The 2^− ΔΔCt^ method was used to calculate relative gene expression [19]. Results were normalized to the 18S rRNA signal. Primer sequences are as follows: PDK1, 5′-GGATTGCCCATATCACGTCTTT-3′ and 5′-TCCCGTAACCCTCTAGGGAATA-3′; LDHA, 5′-ATCTTGACCTACGTGGCTTGGA-3′ and 5′-CCATACAGGCACACTGGAATCTC-3′; BNIP3, 5′-CAGGGCTCCTGGGTAGAACT-3′ and 5′-CTACTCCGTCCAGACTCATGC-3′; P4HA2, 5′-GCCTGCGCTGGAGGACCTTG-3′ and 5′-TGTGCCTGGGTCCAGCCTGT-3′; 18S rRNA, 5′-GAGGATGAGGTGGAACGTGT-3′ and 5′-AGAAGTGACGCAGCCCTCTA-3′.

### Immunoprecipitation and immunoblot assays

Whole cell lysates were prepared in RIPA lysis buffer. 30 μl of protein G-Sepharose beads (GE Healthcare) and immunoprecipitating antibody were incubated with 0.75 mg of cell lysate overnight at 4°C. Beads were washed five times in lysis buffer. Proteins were eluted in SDS sample buffer and separated by SDS-PAGE. Antibodies used in immunoprecipitation and immunoblot assays were: HIF-1α (BD Transduction Laboratory); HIF-1β, TAZ and IgG (Novus Biologicals); and GST and β-actin (Santa Cruz). HRP-conjugated anti-rabbit (Amersham) and anti-mouse (Santa Cruz) secondary antibodies were used. Chemiluminescent signal was developed using ECL Plus (GE Healthcare).

### ChIP assay

ChIP assays were performed as previously described [30]. MCF-7 cells were cross-linked in 3.7% formaldehyde for 10 min and lysed with SDS lysis buffer. Chromatin was sheared by sonication and lysates were pre-cleared with salmon sperm DNA/protein A-agarose slurry (Millipore) and incubated with IgG (Novus Biologicals) or antibodies against the following proteins: HIF-1α (Santa Cruz), HIF-1β and TAZ (Novus Biologicals). Salmon sperm DNA/protein A-agarose slurry was added and the agarose beads were washed sequentially with: low- and high-salt immune complex wash buffers; LiCl immune complex wash buffer; and twice with TE buffer. DNA was eluted in 1% SDS with 0.1 M NaHCO3, and crosslinks were reversed by addition of 0.2 M NaCl. DNA was purified by phenol-chloroform extraction and ethanol precipitation, suspended in 50 μl TE buffer, and a 2-μl aliquot was used for qPCR. The nucleotide sequences of primers used for qPCR are as follows: CTGF, 5′-GGAGTGGTGCGAAGAGGATA-3′ and 5′-GCCAATGAGCTGAATGGAGT-3′; and PDK1, 5′-CGCGTTTGGATTCCGTG-3′ and 5′-CCAGTTATAATCTGCCTTCCCTATTATC-3′.

### GST pull-down assay

GST fusion proteins were expressed in *Escherichia coli* BL21-Gold (DE3) and purified as described [10, 11, 31]. For GST pull-down from cell lysates, equal molar amounts of GST and GST fusion proteins immobilized on glutathione-Sepharose 4B beads were incubated overnight with 2 mg of whole cell lysates. After washing five times, the bound proteins were fractionated by SDS-PAGE, followed by immunoblot assays.

### Luciferase reporter assays

Cells were seeded onto 48-well plates. For p2.1 assays, cells were co-transfected with p2.1 [21] and pSV-Renilla. For GalA assays, cells were co-transfected with pG5E1bLuc and either pGalA or pGalO [22]. For *CTGF* promoter assays, cells were co-transfected with pSV-Renilla, either pGL2-CTGF or pGL2-Basic, and either pcDNA3.1 empty vector or pcDNA3.1-HIF-1α(P402A/P564A) [10]. Transfected cells were exposed to 20% or 1% O_2_ for 24 h. Firefly and Renilla luciferase activities in cell lysates were determined using the Dual-Luciferase Assay System (Promega).

### Statistical analysis

Data are presented as mean ± SEM and were analyzed with an unpaired two-tailed Student's *t* test for two groups or ANOVA followed by Bonferroni post-test for multiple groups. *P* < 0.05 was considered significant.
